# Modelling the transmission dynamics of bovine digital dermatitis in New Zealand pastoral dairy production systems

**DOI:** 10.1186/s13567-020-00750-8

**Published:** 2020-02-21

**Authors:** D. Aaron Yang, Richard A. Laven, Kristina R. Müller, M. Carolyn Gates

**Affiliations:** School of Veterinary Science, Massey University, Palmerston North, 4474 New Zealand

## Abstract

Bovine digital dermatitis (DD) is an important infectious cause of cattle lameness worldwide that has become increasingly prevalent in New Zealand pastoral dairy herds. In this study, a simplified DD scoring system after considering both M and Iowa DD scoring systems was applied to explore the transmission dynamics of DD in a typical spring-calving pastoral New Zealand dairy herd. The modified model only included three compartments: normal skin, early stage lesions and advanced lesions. Lesions regressing after treatment were excluded as DD lesions are rarely treated in New Zealand. Furthermore, sub-classes within each lesion class were not defined due to the lack of variability in DD lesion presentations within New Zealand. The model was validated based on longitudinal field data from three dairy herds in the Waikato region during one lactation season (2017–18). The model suggested that in infected dairy herds, although DD prevalence will tend to increase year-on-year it is likely to remain relatively low (< 18%) even after 10 years of within-herd transmission. It is likely that the low transmission rate during the late lactation (model assumption) results in more cases resolving than developing during this period and therefore results in the low prevalence of infectious cattle at the start of each subsequent lactation. Cattle with advanced lesions had a stronger influence on the establishment and maintenance of DD than cattle with early stage lesions highlighting the importance of targeting these animals for intervention. On-going monitoring of DD is highly recommended to assess the long-term progression of the disease in affected dairy herds.

## Introduction

Bovine digital dermatitis (DD) is an infectious foot disease that causes varying levels of pain, discomfort, and lameness in dairy cattle [1] and has been increasingly found in dairy production systems worldwide [2, 3]. In countries where cows are housed indoors, DD can be a major infectious cause of cattle lameness as well as a significant problem for the dairy industry due to losses in milk production [4], increased treatment costs [5], and negative impacts on animal welfare [6]. Once established in a herd, DD typically becomes endemic and very few herds are able to completely eradicate the disease [7] due to the multifactorial and complex interactions between the bacteria [8], animal [9] and environment [10].

In pasture-based systems, such as those that predominate in New Zealand, DD lesions are typically less commonly seen than in housed cattle [11]. Furthermore, clinical lameness was rarely associated with DD under New Zealand conditions [12], it is therefore likely that DD will have only limited impacts on herd-level production [13]. However, routine monitoring of DD is still recommended to identify early cases of the disease and to make sure DD remains manageable at the herd level [14].

From an epidemiological perspective, recording the different morphological stages of DD lesions in cattle is also important as it provides insights into the pathophysiology of the disease [15] and its transmission dynamics at the population level [16]. The most widely used classification system for DD lesions is the “M score” scheme developed by Döpfer et al. [17] and extended by Berry et al. [18]. The description of each “M score” is summarised in Table 1.

**Table 1:** Description of different stages of bovine digital dermatitis lesion using “M scores”.

M-score	Descriptor
M0	Normal skin
M1	Early stage, small (< 2 cm) focal active state. The surface is moist and ragged with mottled red-grey
M2	Classical ulcerative active stage, usually large (> 2 cm across). Painful upon palpation
M3	Healing stage after antibiotic treatment. The ulcerated surface is covered by dry black scab
M4	Chronic stage. The hyperkeratotic lesion can have a proliferative aspect
M4.1	Chronic stage with small active M1 focus

As described by Döpfer et al. [17] and adapted by Berry et al. [18].

Two previous studies [16, 19] have built mathematical models to explore the lesion transition dynamics of DD in a housed cow setting. Döpfer et al. [16] adopted an SEIS structure to describe the lesions transitions in a large, closed population of dairy cattle. They assumed that class M0 was the susceptible class (S), that classes M1 and M3 were latently infected or exposed (E) and that classes M2 and M4 were infectious (I). The model restricted the potential transitions between stages (e.g. M2 could be moved in from both M1 and M4, but could only move out to M3 and M3 could not move back to M2). In contrast, Biemans et al. [19] considered that all classes except M0 were infectious, and adopted the SIS structure to study the relative contributions of different M classes to DD transmission. They did not restrict the potential transitions between stages. Döpfer et al. [16] identified that the speed of identifying acute lesions (M2) and the effectiveness of treating these lesions were the keys to DD control. In contrast, Biemans et al. [19] concluded that M4 lesions made the greatest contribution to disease transmission and that control should be focused on lowering the number of M4 lesions.

Although these models provide valuable insights, model frameworks based on “M scores” are difficult to implement in New Zealand dairy herds due to differences in DD lesion presentations and the limitations of DD inspection during milking without lifting the feet. Most affected cattle in New Zealand have M4-like lesions. These are typically small grey, rubbery lesions which may or may not have thickened, darker edges, although larger, papillomatous lesions can also be found with much lower frequency. Red active (M2) lesions are extremely rare [20]. Post-treatment M3 lesions are not a feature of the disease in New Zealand as lesions are treated only very rarely. Herd size and lack of suitable facilities means that on most farms the only feasible method of DD detection is observation during milking, ruling out accurate identification of M1 and M4.1 stages [21]. While the current models based on the “M scores” include all lesion types [16, 19], it is not necessary to adopt either model framework to describe a much simpler situation where the lesion presentations are lack of variation. Thus, there is a need to explore a simpler classification schemes when creating transmission models that are designed for pasture-based production system. In addition, models based on the “M score” did not focus on the differences between these M4-like lesions, however, we hypothesized that the large papillomatous lesions is likely to have different impact on the within-herd prevalence in a long term compared to the small grey, rubbery lesions.

One alternative to the “M scores” scheme is the Iowa DD scoring system [22]. According to this system, there are six stages that are normal skin (stage 0), lesion onset (stage 1), developing lesions (stage 2), ulcerative lesions (stage 3), chronic lesions (stage 4) and lesions after treatment (stage 5). Stage 1 and stage 2 are both considered as early lesions and they are further subdivided into type A (non-proliferative dermal pittings or advanced erosive, proliferative lesions located within the interdigital cleft, corresponding to A1 and A2, respectively) and type B lesions (focal or multifocal proliferative scabs on heel or proliferative scabs distributing across heel diffusely, corresponding to B1 and B2, respectively).

Both aforementioned DD scoring systems are far more difficult to describe the current New Zealand DD presentations, however, lesions in New Zealand can be at least partially described using either system, i.e. a dyskeratotic lesion located within the interdigital fold is corresponding to the stages A1 and A2 using the Iowa DD scoring system and M4 in the “M scores” [18, 22]. However, lack of variations of lesion presentations requires that the classification needs to be simplified to fit New Zealand conditions.

The aim of this study was to use a simplified DD lesion scoring system to explore the transmission dynamics of DD under New Zealand pastoral production systems. The validity of the simulation model was assessed by comparing the model output with the longitudinal field data collected from DD affected herds across a single lactation. The implications for disease control based on the model findings are also discussed.

## Materials and methods

### Field data

Longitudinal data on lesion occurrence and type were collected over a single lactation season in three spring-calving herds in the Waikato region of New Zealand. The herds had all been previously identified as having DD from a previous cross-sectional study [20] and were selected on a convenience basis since they were located close together, the farmers were willing to have repeated visits from the researcher, and the farmers agreed not to use DD treatments during the study (unless there was a significant welfare concern for the animal). Only Holstein–Friesian and Holstein–Friesian cross cows were raised in all these herds and they were milked twice a day in herringbone milking parlours. The herds were intended to be visited weekly by the first author over the 36 weeks period from 21^st^ August 2017 to 30^th^ April 2018. However, due to scheduling conflicts, a total of 19 observation weeks were available for Herd 1, 16 observation weeks for Herd 2, and 17 observation weeks for Herd 3. The average observation interval between two visits was 2 weeks, with the minimum observation interval being 1 week while the maximum observation interval being 7 weeks.

Each herd visit was timed during the day to coincide with routine milking. The rear feet of all milking cows were hosed as necessary to remove mud and faecal contamination before being examined with an aid of a hand torch to identify DD lesions [23]. The photos of the lesions were taken, and short descriptions of their appearances were noted for further categorizing purpose. The maximum numbers of cows examined at a milking were 286, 194, and 273 for Herd 1, Herd 2 and Herd 3, respectively. The aim of collecting the field data was to calibrate the simulation model which was used to investigate the prevalence patterns in a long term.

### Simulation model

A simple deterministic compartmental model was then developed to capture the transmission dynamics of DD in a typical spring-calving pastoral New Zealand dairy herd. This included a demographic component and disease component as described below.

#### Herd demographics

A simplified herd demographic structure was modelled based on the typical annual management calendar of a spring-calving dairy herd in New Zealand. The start of lactation was set to 1^st^ Aug and lasted 253 days until the fixed herd dry-off date of 10^th^ April the following year (this setting was to match the field observation period). Culling was modelled using a simplified assumption that 20% of the cows would be culled on a single day (set on 23^rd^ April) towards the end of lactation. The dry period lasted from 11^th^ April to 31^st^ July. After 31^st^ July, a new lactation season started and the replacement animals joined the herd on the first day of lactation. The replacement rate was set to be equal to the culling rate (20%) to make sure the herd size remained constant over time. The above process was repeated for each year until the end of the simulation.

#### Disease dynamics

**Figure 1:**
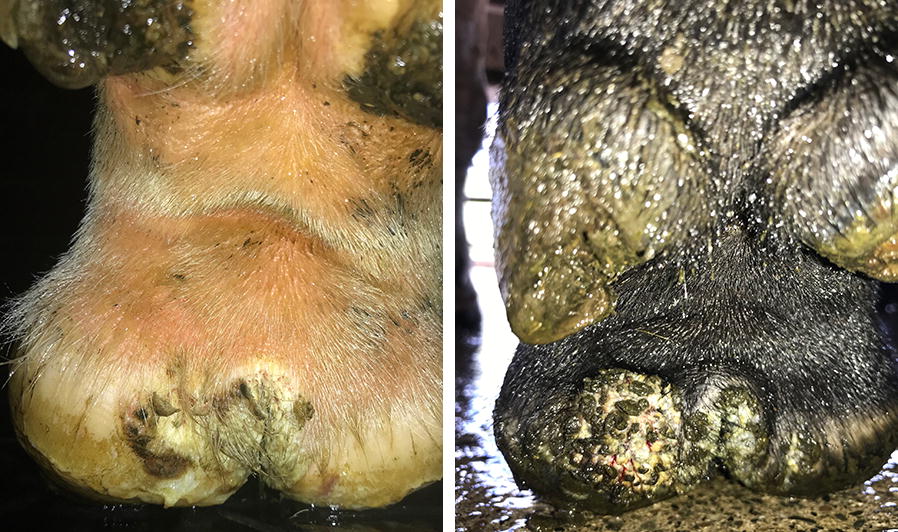
**Early (left) and advanced (right) digital dermatitis lesions observed in New Zealand dairy herds.** The foot that has an advanced lesion (papillomatous on the left heel) also has an early lesion (circumscribed hyperkeratosis that begins in the interdigital space). If both an advanced lesion and an early stage lesion were diagnosed on the same foot, the lesion would be recorded as an advanced lesion.

**Figure 2:**
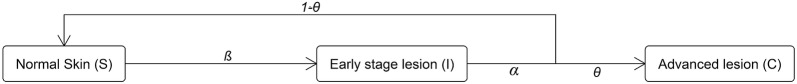
**Simplified digital dermatitis lesion development model based on Krull et al. [**22**].***β*: transmission coefficient, *α*: the reciprocal of the average time that early stage lesions persist, *θ*: probability that an early stage lesions progresses to an advanced lesion.

The types of lesions were defined as following. An early stage lesion is a dyskeratotic lesion located within the interdigital fold, which corresponds to the stages A1 and A2 using the Iowa DD scoring system and M4 in the “M scores”. An advanced lesions is a large hyperkeratotic lesion with filamentous proliferative skin alteration which could be regarded as a stage 4 lesion according to Iowa DD scoring system or M4 using the “M scores” (summarised in Figure 1). If both an advanced lesion and an early stage lesion were diagnosed on the same foot, the lesion would be recorded as an advanced lesion. The transitions between these two DD lesion types is described in Figure 2 similar to Iowa DD lesion development model except that the compartment for regressing lesions after treatment was excluded [22]. A susceptible animal $\left( {\text{S}} \right)$ was assumed to get infected at a transmission rate $\beta$. Once infected, the animal could develop an early stage lesion $\left( {\text{I}} \right)$ that was assumed to persist for an average of certain days leading to a transition rate out of the compartment of α—the reciprocal of the persistence duration, before regressing to a susceptible state. Alternatively, an early stage lesion might progress to an advanced lesion $\left( {\text{C}} \right)$ with a probability $\theta$ and an advanced lesion would persist. Additional assumption was made that both lesion types were infectious and had the same infectivity.

It was assumed that there was homogeneous mixing of individual cows and a constant group size. This meant that the transmission coefficient ($\beta$), the product of the contact rate between animals and the probability of the contact leading to infection, was the same for all susceptible animals [24]. Disease transmission was assumed to be most rapid in early lactation [25]. Therefore, $\beta$ was time-dependent, such that $\beta_{t} = \beta_{t - 1} - \delta$, where $\delta$ reflected the difference between the transmission coefficients between day $t - 1$ and day $t$ during the lactation, and was assumed to be constant. The probability that the contacts were with infectious animals ($p$) was modelled as a frequency-dependent transmission, $\frac{{{\text{I}} + {\text{C}}}}{\text{N}}$ [24], where ${\text{N}}$ was the total number of cows in the population.

In the deterministic form, changes in the number of animals in each compartment were modelled using the following equations:

1 $${\text{S}}_{t} = {\text{S}}_{t - 1} - \frac{{\beta_{t - 1} {\text{S}}_{t - 1} \left( {{\text{I}}_{t - 1} + {\text{C}}_{t - 1} } \right)}}{{{\text{N}}_{t - 1} }} + \alpha {\text{I}}_{t - 1} \left( {1 - \theta } \right),$$

2 $${\text{I}}_{t} = {\text{I}}_{t - 1} + \frac{{\beta_{t - 1} {\text{S}}_{t - 1} \left( {{\text{I}}_{t - 1} + {\text{C}}_{t - 1} } \right)}}{{{\text{N}}_{t - 1} }} - \alpha {\text{I}}_{t - 1} ,$$

3 $${\text{C}}_{t} = {\text{C}}_{t - 1} + \alpha {\text{I}}_{t - 1} \theta .$$

#### Model validation and calibration

The structure of the simulation model was firstly validated by comparing with the field observation data. During the data collection, transitions between normal skin and early stage lesions were frequently observed, which agreed with the model. We also observed no transitions from the only advanced lesion recorded in this study, and its size and appearance did not change appreciably between observations. These observations suggest, in agreement with the model that the advanced lesion may represent a chronic form of DD in New Zealand dairy cattle. However, we did not observe any transitions to an advanced lesion. This is consistent with the findings of Krull et al. [22] that early stage lesions did not have to progress to the advanced stage, but could remain as early lesions for a certain period. We therefore assumed that the advanced lesion observed had developed before the beginning of data collection and was preceded by an early stage lesion [22].

As far as the authors’ awareness, the DD lesion classification system developed in Iowa [22] has not previously been used for simulation modelling purposes, therefore, no references are available to provide estimates for the parameters required in such model. Hence, initial parameter values were estimated based on the authors’ opinion or field observations from New Zealand. The value for the transmission coefficient for the first day of lactation ($\beta_{{t_{0} }}$) was initially set to be 0.055 as our best estimate. Since we assumed the transmission rate decreased over time by a fixed constant, we initially set $\delta = 0.0002$. The transition rate ($\alpha = 0.0202$) moving from an early stage lesion to other compartments was the reciprocal of the average persistence of an early stage lesion, which was approximately 7 weeks (~49 days) based on field observations of this study. The probability $\left( \theta \right)$ that an early stage lesion would progress to an advanced lesion was expected to be extremely low, however the exact value was difficult to determine. To be conservative, we used a small value of 0.2% for $\theta$ and we carried out a sensitivity analysis to assess the impact of different values of $\theta$ on the model output.

Additional calibration of the values for $\beta_{{t_{0} }}$, $\alpha$ and $\delta$ was performed by comparing the seasonal pattern predicted by the simulation model with the field data. The field data were analysed using the following steps: first, the scatter plot of observed prevalence of DD in the three herds against time was plotted. Based on the plot, a generalised linear mixed model (GLMM) treating “herd” as a random effect and time $\left( X \right)$ and $X^{2}$ as fixed effects were constructed with a binomial distribution and logit link function. The variance of the random effect was extremely small, confirming the homogeneity of the herds. The model was therefore re-constructed using a generalised linear model (GLM) with a binomial distribution and logit link function. This process was performed using Stata 13 (StataCorp, USA). Based on the estimated regression coefficients, a fitted curve was obtained to compare the field data to the predicted prevalence curve from the simulation model so that the fit of the simulation model could be visually assessed. To ensure a good fit, the values for $\beta_{{t_{0} }}$, $\alpha$ and $\delta$ were manually calibrated until the predicted curve from the simulation model aligned with the fitted curve by the GLM. Table 2 summarises the parameters required in the model and their corresponding values.

**Table 2:** The parameters and their corresponding values used in the simulation model

Parameter	Value	Descriptor
*β*_*t0*_	0.0552	The transmission rate at the first day of lactation of every year in the model (1^st^ August)
*δ*	0.00022	Deviation between the transmission rates of two consecutive days over a lactation season
*α*	0.0202	Reciprocal of the time that an early stage lesion persists
*θ*	0.002	Probability that an early stage lesion transits to an advanced lesion

#### Simulation conditions

The calibrated simulation model was then used to explore the transmission dynamics of DD in a population of dairy cows in New Zealand over a period of 10 years. The time step of the model was 1 day. To capture the low prevalence of DD lesions observed in the field, the herd size needed to be large and was therefore set at 1000. For the first model, a single animal with an advanced lesion was assumed to be in the population at the beginning of the simulation representing a chronically infected animal introduced at the start of the first lactation (scenario A). The total animals in the population of each simulation day were monitored to make sure the demographics stabilised. The primary outputs of the model were: the long term seasonal pattern of disease, including the timing and the level of the peak prevalence in each year.

Three other initial scenarios were modelled to establish whether the early stage or advanced lesions contributed more to the establishment of DD on New Zealand dairy farms: (B) one animal with an advanced lesion and one animal with an early stage lesion; (C) one animal with an early stage lesion; and (D) two animals with early stage lesions at the beginning of the first lactation.

#### Sensitivity analysis

A sensitivity analysis was carried out to examine the impact of changing $\alpha$ and $\theta$ on the simulation outputs. The sensitivity analysis scenarios are summarised in Table 3.

**Table 3:** Different scenarios tested in the sensitivity analysis for the simulation model

Parameter	Sensitivity analysis scenarios			
	Scenario 1	Scenario 2	Scenario 3	Scenario 4
*α*	0.0238	0.0178	0.0202	0.0202
*θ*	0.002	0.002	0.001	0.003

*α*: Reciprocal of the time that an early stage lesion persists; *θ*: Probability that an early stage lesion transits to an advanced lesion.

### Statistical analysis

The simulation outputs with each initial infection conditions were plotted against time for a ten year period. The peak prevalences reached during each of those 10 years were summarised for each of the four scenarios. For the scenarios where the advanced lesion was absent at the beginning (scenario C and D), the year that the first advanced lesion was observed was identified.

## Results

### Model validation

**Figure 3:**
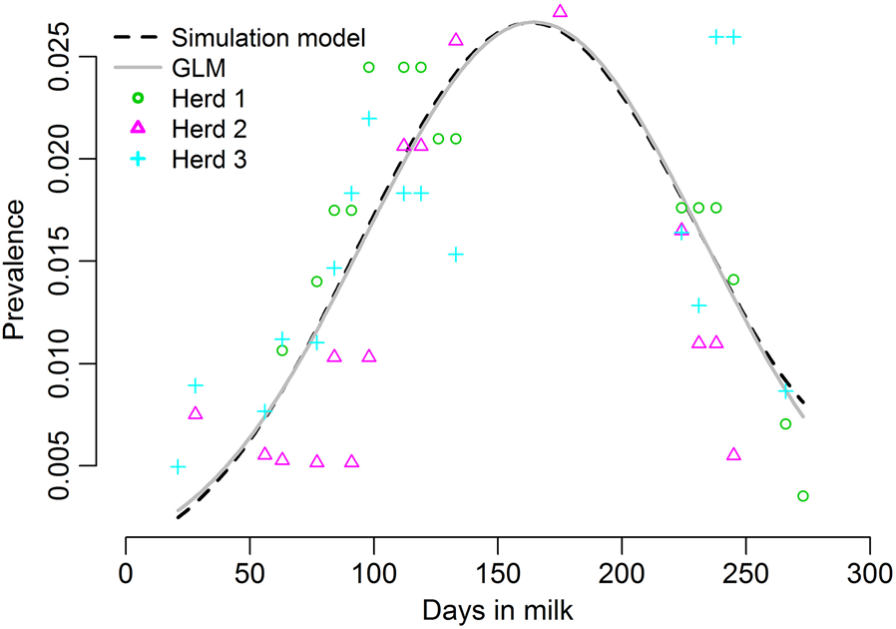
**Comparing the simulation model output to the field observations.** The simulation model is valid as its predicted seasonal pattern (red dashed curve) agrees with the fitted seasonal pattern (blue solid curve) from the filed data collected in three New Zealand dairy herds in 2017–18 lactation season using the generalised linear model (GLM).

As shown in Figure 3, there was close agreement between the simulation model and field data indicating adequate model fit. In the 2017–2018 lactation seasons, the peak prevalence was 2.7% on 11^th^ January 2018.

### DD dynamics

**Figure 4:**
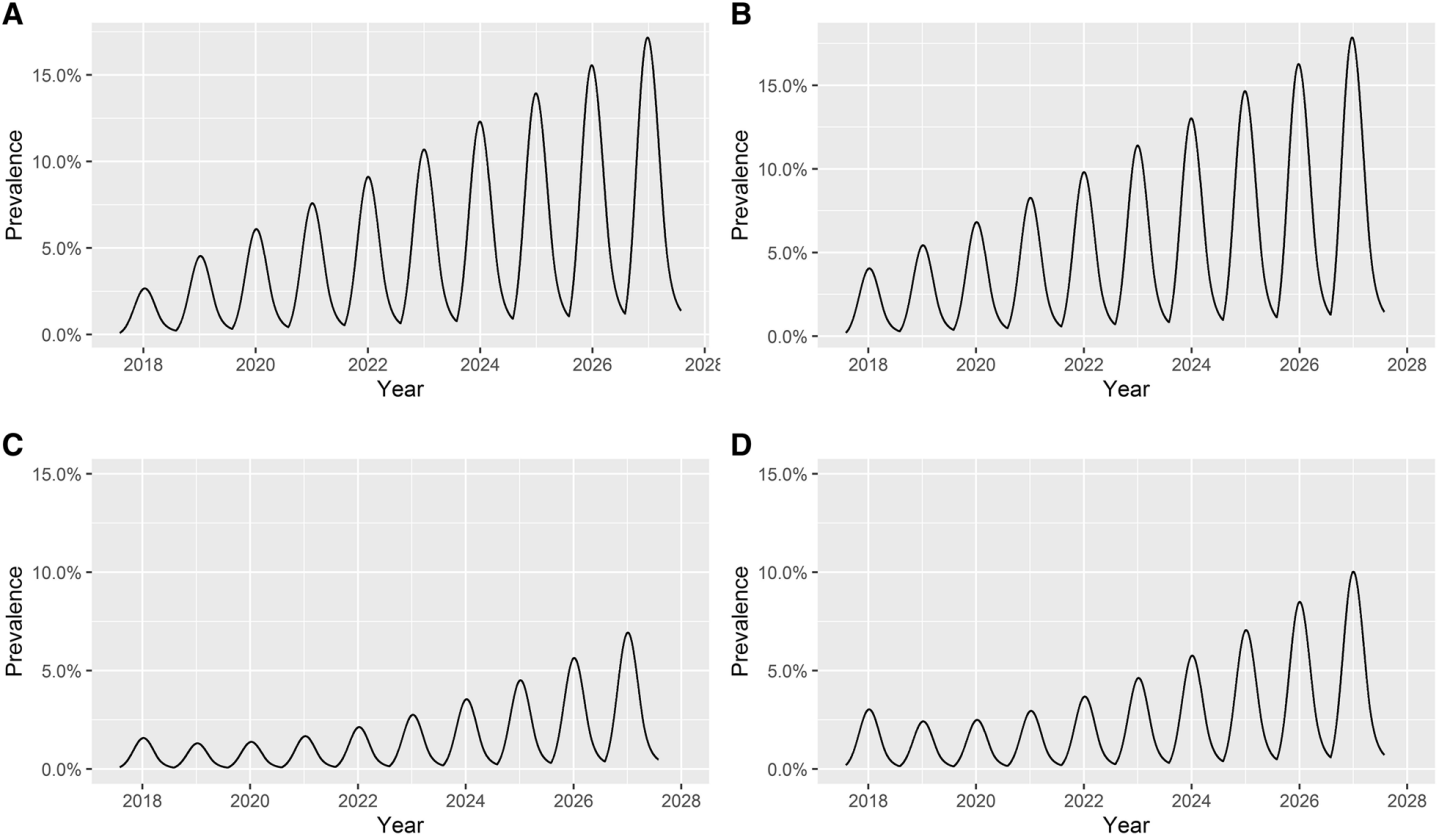
**Predicted seasonal patterns of digital dermatitis for 10** **years with different initial lesion distributions. A** one advanced lesion, **B** one advanced lesions & one early stage lesion, **C** one early stage lesion, **D** two early stage lesions.

Based on the model predictions, given the presence of one advanced lesion at the start of lactation, DD prevalence would increase to a peak in mid-lactation and then decrease during the late lactation and dry periods. However, DD prevalence at the start of each subsequent season would be higher than that of the previous season. Thus DD prevalence will increase year-on-year. The dynamics of DD at the herd level were different depending on whether an advanced or an early stage lesion was assumed to be present at the beginning of the first lactation of the modelling period. As per Figure 4, if an advanced lesion was found in the first year, DD prevalence would continue to increase in the subsequent 10 years (Figure 4, panels A and B). However, if advanced lesions were absent at the beginning of the lactation in the first year (Figure 4, panels C and D), then DD prevalence declined in the second year and remained at a similar level in the third year before starting to increase from the fourth year.

In addition to the impact on the dynamics of DD, the different lesion types also had different effects on the establishment of DD in a herd. By comparing panels “A” to “B” of Figure 4, it was clear that given the existence of an advanced lesion at the beginning of the first lactation, the presence of early stage lesions did not speed up DD transmission. After 10 years, the peak prevalences of the two different initial infection scenarios were 17.2% and 17.9%, respectively. If there were no advanced lesions at the beginning of the first lactation of the 10-year modelling period (panels C and D of Figure 4), then DD prevalence would only go up to 7% over 10 years if one early stage lesion was found at the beginning of the first lactation, and 10% if two lesions were found.

The number of early stage lesions at the beginning of the first lactation of the modelling period influenced the speed of establishment of the first advanced lesion in the herd. The model suggested that it would take almost 9 years to develop the first advanced lesion if there was only one early stage lesion found at the beginning of the first lactation, whereas the first advanced lesion was seen in early lactation in the sixth year if two early stage lesions were found at the beginning of the first lactation.

### Sensitivity analysis

**Figure 5:**
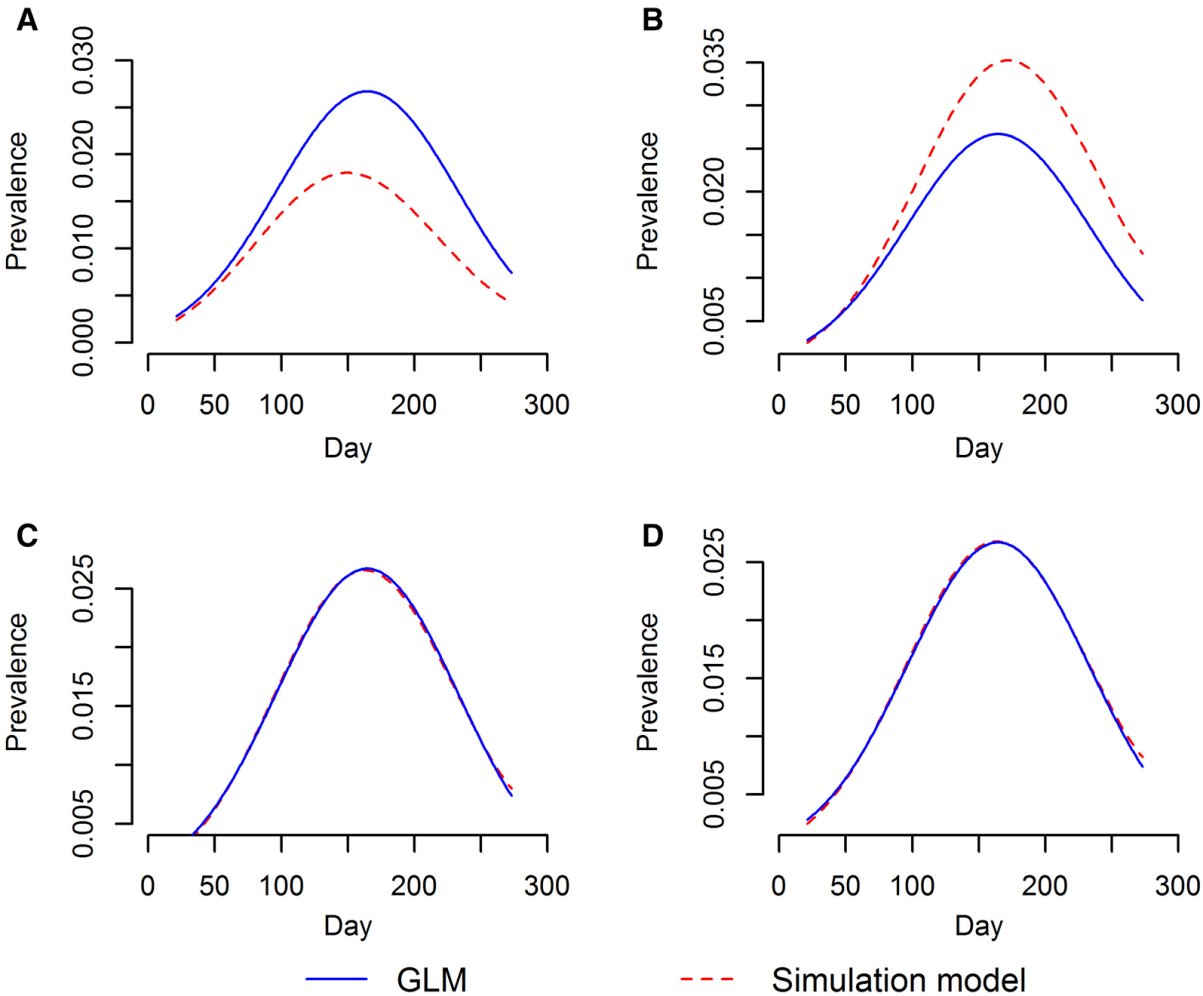
**Results of sensitivity analysis with different values for*α*and*θ.****α*: the reciprocal of the average time that early stage lesions persist, *θ*: probability that an early stage lesions progresses to an advanced lesion; **A***α* = 0.0238 animal/day and *θ* = 0.002, **B**: *α* = 0.0178 animal/day and *θ* = 0.002, **C***α* = 0.0202 animal/day and *θ* = 0.001, **D***α* = 0.0202 animal/day and *θ* = 0.003.

The results of the sensitivity analysis are displayed in Figure 5. Increasing or decreasing the infectious duration of the early stage lesion ($\alpha )$ by a week (7 days) both had obvious influences on the simulation model’s output (Figure 5, panels A and B). In contrast, altering the probability of an early stage lesion progressing to an advanced lesion $\left( \theta \right)$ had minimum impact on the simulation outputs, the model still had reasonably good fit if $\theta$ was changed to 0.3% or 0.1% (Figure 5, panels C and D).

## Discussion

The simulation model based on the simplified DD scoring system was validated and calibrated based on longitudinal field observations from three herds during the 2017–2018 lactation season. According to the model, the prevalence of DD in affected herds was predicted to remain relatively low even after 10 years of within-herd transmission. This, according to the model, is most likely to because the transmission parameter decreases after calving, which results in a more lesions resolving than developing during the late lactation and dry period, thereby reducing the infection burden in the at the start of the following lactation. This is consistent with data from non-pasture-based systems where dry cows are at lower risk of having DD than lactating cows [26–28]. While in the pasture-based system, climate factors such as monthly rainfall level and soil temperature could also have impact on the prevalence of DD, therefore having lower prevalence during the late lactation and dry period is also likely due to the differences of climate [29]. The model suggests that on New Zealand dairy farms which have DD, the low within-herd prevalence of DD is likely to persist for many years, making it uneconomic for farmers to implement control measures such as routine footbaths which are commonly used to control BDD in housed cattle [30, 31]. However the model did suggest that disease prevalence was likely to continue to increase year-on-year. Therefore, we recommend that on infected farms, New Zealand dairy farmers should undertake on-going monitoring of DD to assess the progress of the disease. Ideally, monitoring should occur multiple times during the season as part of routine foot health and lameness assessments [32]. However, if farmers can only conduct DD monitoring at a single time point, the model results suggest that late November to end of February would perhaps be the optimal time as the DD prevalence is likely to reach its peak during this period. This suggestion is consistent with the seasonality seen in a previous New Zealand-based longitudinal study [29].

During the monitoring process, it is important for observers to differentiate between the different lesion types as the relative prevalence of cattle with early stage and advanced lesions can change the transmission dynamics, in particular the speed of increase of peak prevalence. The model predicts that if advanced lesions are present in only 0.1% of cattle then in 10 years’ time the prevalence of DD will be ~17% whereas if that 0.1% has only early stage lesions then the equivalent figure will be 7%. Thus the model results highlight the potential importance of advanced lesions in the establishment of DD in New Zealand dairy herds. Nevertheless, early stage lesions cannot be ignored completely as increasing the number of early stage lesions present at the start of the modelling period decreases the time prior to the development of the first advanced lesion, thereby increasing the peak prevalence of DD seen 10 years later.

Advanced lesions are very rare in New Zealand. So far, out of 1353 DD affected cows detected by the first author over the years, four “advanced lesions” including the one found in this study have been observed [11, 20]. The other three which are morphologically similar to the advanced lesion found in this study were only observed once in cross-sectional studies and no further observations were made on those three. Thus we lack data on the factors that determine the proportion of lesions which become advanced lesions. However, North American experience suggests that three key factors influence the progression from an early stage lesion to an advanced lesion: (1) presence of necessary bacterial agents, (2) cattle genetics and immunity, and (3) environment [22, 33]. For example, pasture-based system provides cleaner environment for dairy cows compared to the confined system. Further research is needed to establish whether these key factors are important in the development of advanced lesions in New Zealand pastoral dairy herds.

This study clearly demonstrated that the simulation model created on the simplified DD scoring system could accurately describe lesion progression and regression in the natural condition in New Zealand dairy herds. And the simplified DD scoring system used in the development of this model would be relatively simple for veterinary technicians or farmers to implement to identify the different types of DD lesions in pasture-based system such as New Zealand. However, further research is needed to confirm the within- and between-observer repeatability of this system [34–36].

As with any simulation model, there are limitations that may affect interpretation of the results. Firstly, we were unable to generate precise estimates for the average length of time cows spent in each lesion state because it was not possible to observe animals more frequently than once per week and, due to schedule conflicts, the time period between consecutive observations was sometimes longer (maximum observation interval was up to 7 weeks). This is important as there was a relatively large impact of changing the values for α in the sensitivity analysis. In future studies, it may be beneficial to perform more intensive observations in affected herds by using multiple observers, though this will require validation of the scoring system and on-going training [21, 36]. Secondly, this model adopted simplified herd demographics without considering the calving and culling patterns in the three herds. This could affect the within-herd transmission dynamics if, for example, in the future, cows with DD lesions are more likely to be culled. Future studies should collect these data along with the DD lesion data. Thirdly, as the data used to calibrate this simulation model were only obtained from three herds in a single lactation season, its predictive ability is worth discussing. Previous research has identified that under New Zealand conditions, climate has a significant effect on BDD prevalence [29]. Therefore, it is likely that if there are changes in the climate of New Zealand due to climate change (such as wetter winters and drier summers), this model may no longer be predictive (as it is based on the current climate). Thus if there is climate change we suggest that this model be revisited and updated.

Currently, DD lesions have not been observed in the vast majority of dairy cattle in New Zealand. This situation could persist for many years. However the model suggests that disease prevalence is likely to increase consistently in the future, increasing the chance that DD will become clinically significant. Thus we suggest that on-going monitoring of DD should be undertaken to assess the progress of the disease, and that this should pay attention to both lesion types but particularly the advanced lesions.

## Data Availability

The data collected in the current study are not publicly available as they contain confidential information of the participated farmers. However, the datasets are available from the corresponding author on reasonable request.

## Abbreviations

DD: Digital dermatitis
GLMM: Generalised linear mixed model
GLM: Generalised linear model
